# Induced Pluripotent Stem Cells in Corneal Regeneration: Biological Progress, Translational Barriers and Clinical Outlook

**DOI:** 10.3390/biomedicines14061323

**Published:** 2026-06-11

**Authors:** Tareq S. Al-amarat, Jodhbir S. Mehta

**Affiliations:** Singapore National Eye Centre, Singapore 168751, Singapore; tareq.sulaiman.i.a@singhealth.com.sg

**Keywords:** induced pluripotent stem cells (iPSCs), corneal regeneration, limbal stem cell deficiency, corneal epithelium, corneal endothelium, corneal stroma, regenerative medicine, cell therapy

## Abstract

Corneal blindness remains a major cause of visual impairment worldwide and may result from trauma, infectious keratitis, degenerative disorders, endothelial dysfunction, and limbal stem cell deficiency (LSCD). Although corneal transplantation remains the standard treatment for advanced disease, its effectiveness is limited by donor tissue shortage, immune-mediated rejection, postoperative complications, and progressive graft failure. These limitations have accelerated interest in regenerative approaches aimed at restoring native corneal structure and function. Induced pluripotent stem cells (iPSCs) have emerged as a promising platform for corneal regeneration because of their pluripotency, self-renewal capacity, and potential for autologous or immune-compatible therapy. Recent advances in differentiation protocols have enabled the generation of corneal epithelial-like cells, stromal keratocyte-like cells, and corneal endothelial-like cells from iPSCs. Preclinical studies have demonstrated encouraging improvements in corneal transparency, epithelial restoration, fibrosis reduction, and endothelial function, while early clinical investigations, particularly in LSCD, have reported favorable short-term safety and functional outcomes. However, major translational barriers remain, including tumorigenicity, immunogenicity, genomic instability, manufacturing complexity, scalability, and long-term safety concerns. Stromal regeneration also remains comparatively underdeveloped relative to epithelial and endothelial applications. This review summarizes current differentiation strategies, biological mechanisms, preclinical and early clinical evidence, and the principal translational challenges associated with iPSC-based corneal regeneration. Overall, iPSC-derived corneal therapies demonstrate considerable regenerative potential, although further standardization, long-term safety evaluation, and multicenter clinical validation remain necessary before widespread clinical adoption.

## 1. Introduction

The cornea is a highly specialized, transparent, and avascular tissue that forms the anterior surface of the eye and contributes approximately two-thirds of its refractive power. Consequently, even minor disruptions in corneal architecture or cellular homeostasis may result in significant visual impairment. Corneal injury arising from hereditary conditions, trauma, infection, chemical exposure, autoimmune disease, or limbal stem cell deficiency (LSCD) frequently leads to scarring, neovascularization, stromal opacification and irreversible vision loss. Globally, corneal disease remains one of the leading causes of blindness, with a disproportionate burden in low- and middle-income regions where access to donor tissue and specialized ophthalmic care remains limited [1].

Advances in surgical management, particularly the widespread adoption of lamellar keratoplasty techniques, have improved visual outcomes and reduced complication rates compared with traditional penetrating keratoplasty. However, corneal transplantation continues to face substantial limitations, including donor shortages, immunologic rejection, postoperative complications, endothelial cell loss, and progressive graft failure over time. Endothelial cell attrition, in particular, remains a major determinant of long-term graft survival and functional decline following transplantation [2]. These limitations have driven growing interest in regenerative strategies aimed at restoring native corneal structure and function rather than relying exclusively on donor tissue replacement.

The development of induced pluripotent stem cell (iPSC) technology by Takahashi and Yamanaka [3,4] represented a major advance in regenerative medicine. Through somatic cell reprogramming, iPSCs can acquire pluripotent characteristics while avoiding many of the ethical concerns associated with embryonic stem cells. In addition, their theoretically unlimited self-renewal capacity and potential for patient-specific or immune-compatible therapies make them particularly attractive for ocular surface reconstruction and corneal bioengineering. For corneal applications, iPSCs are particularly attractive, as they provide a single platform capable of generating all major corneal cell lineages. As a result, iPSC-based strategies have become central to next-generation approaches for corneal regeneration, although their translation to routine clinical practice remains challenging.

This review summarizes the current biological principles, differentiation strategies, preclinical evidence, early clinical applications, and major translational barriers associated with iPSC-based corneal regeneration, with emphasis on the strengths, current limitations, and future clinical potential of these emerging therapies.

## 2. Corneal Structure and Cellular Requirements for Regeneration

Effective corneal regeneration requires a detailed understanding of corneal anatomy, cellular specialization, and tissue-specific functional requirements [5]. The cornea consists of three principal layers—the epithelium, stroma, and endothelium—each of which performs distinct and highly coordinated functions essential for maintaining transparency and visual quality. Consequently, successful regenerative strategies must address not only cellular replacement but also restoration of tissue architecture and physiological function.

The corneal epithelium is a stratified squamous layer that provides both a protective barrier against environmental insults and a smooth refractive surface. Its long-term homeostasis depends on limbal stem cells located at the corneoscleral junction. Loss or dysfunction of these cells results in limbal stem cell deficiency (LSCD), a condition characterized by epithelial instability, chronic inflammation, conjunctivalization, neovascularization, and progressive visual impairment. The clinical success of autologous limbal stem cell transplantation highlights the importance of restoring a functional stem cell niche for sustained epithelial regeneration [6]. However, although this approach is effective in unilateral disease, bilateral LSCD remains particularly challenging because of the absence of healthy autologous limbal tissue. Alternative autologous epithelial sources, including cultivated oral mucosal epithelial transplantation, have therefore been investigated. Despite encouraging clinical outcomes in selected cases, these approaches do not fully replicate the structural, biological, and optical characteristics of native corneal epithelium. As a result, increasing attention has focused on induced pluripotent stem cell (iPSC)-derived corneal epithelial cells as a potentially patient-specific and scalable regenerative strategy.

The corneal stroma accounts for approximately 90% of corneal thickness and consists of highly organized collagen lamellae maintained by quiescent keratocytes. Preservation of stromal ultrastructure is critical for corneal transparency. Following injury, keratocytes may differentiate into fibroblasts and myofibroblasts, resulting in extracellular matrix disorganization, fibrosis, and stromal haze. Stromal regeneration therefore represents a particularly complex challenge, as successful restoration requires not only cellular repopulation but also precise reconstruction of extracellular matrix architecture and collagen alignment. Despite advances in iPSC-derived keratocyte differentiation, convincing long-term in vivo evidence demonstrating restoration of native stromal organization remains limited.

**Figure 1:**
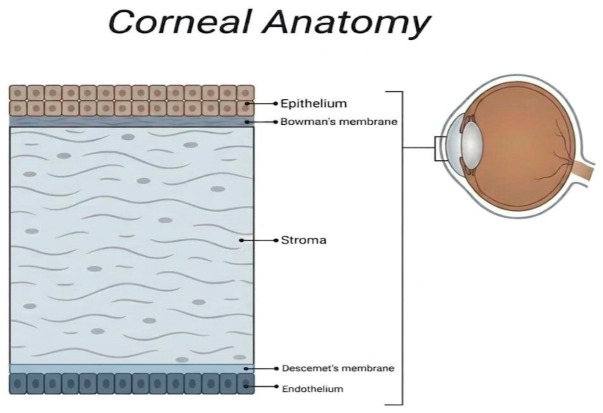
Schematic illustration of normal corneal anatomy, including the corneal epithelium, stroma, endothelium. Each corneal compartment plays a critical role in maintaining corneal transparency and visual function.

The corneal endothelium is a monolayer of hexagonal cells responsible for maintaining stromal deturgescence through active ion transport and barrier function. Because human corneal endothelial cells exhibit minimal proliferative capacity in vivo, endothelial dysfunction remains one of the leading indications for corneal transplantation worldwide. Consequently, regenerative approaches targeting the endothelium must restore both adequate endothelial cell density and long-term functional pump activity to achieve durable corneal clarity. The major anatomical layers of the cornea and their structural organization are illustrated in Figure 1.

## 3. Differentiation of iPSCs into Corneal Cell Lineages

### 3.1. Differentiation Strategies

Most differentiation protocols aim to recapitulate key stages of ocular embryogenesis. In general, induced pluripotent stem cells (iPSCs) are initially directed toward neural ectodermal or neural crest–like intermediates, followed by exposure to lineage-specific growth factors, transcriptional regulators, and small-molecule signaling modulators [7,8]. Recent efforts have increasingly focused on feeder-free, serum-free, and xeno-free culture systems to improve reproducibility and facilitate compliance with good manufacturing practice (GMP) requirements for clinical translation [9].

Despite substantial advances, differentiation efficiency, phenotypic stability, and functional maturity remain inconsistent across protocols and iPSC lines. Variability in donor source, reprogramming strategy, culture conditions, and differentiation timelines contributes to significant inter-study heterogeneity. In addition, residual epigenetic memory and batch-to-batch inconsistencies may influence lineage commitment and long-term cellular behavior. These limitations continue to complicate protocol standardization and highlight the need for validated potency assays, reproducible manufacturing pipelines, and consensus criteria defining corneal cell identity and functional maturity.

### 3.2. iPSC-Derived Corneal Cell Types

iPSC-derived corneal epithelial-like cells have demonstrated the ability to stratify, establish barrier function, and express canonical epithelial markers such as KRT3 and KRT12, supporting their use in limbal stem cell deficiency (LSCD) models and early clinical investigations [7,8,10]. Several studies have also reported partial restoration of ocular surface integrity and improvement in epithelial stability following transplantation. However, differences in differentiation efficiency, epithelial maturation, and long-term engraftment continue to limit direct comparison across studies.

iPSC-derived stromal keratocyte-like cells have shown the capacity to produce organized extracellular matrix components and may exhibit lower profibrotic activity compared with activated corneal fibroblasts. Nevertheless, stromal regeneration remains comparatively underdeveloped relative to epithelial and endothelial applications. Maintaining a stable keratocyte phenotype during prolonged culture remains difficult, and convincing in vivo evidence demonstrating restoration of native stromal ultrastructure and transparency remains limited [11]. In addition, the complex collagen lamellar organization required for optical clarity has proven challenging to reproduce using current bioengineering approaches.

Similarly, iPSC-derived corneal endothelial-like cells express pump-associated markers and demonstrate barrier-related functional properties in vitro. Early preclinical and first-in-human studies have reported encouraging short-term outcomes following endothelial cell transplantation. However, important concerns persist regarding long-term functional durability, cellular maturity, immunogenicity, and safety following transplantation, particularly given the limited proliferative reserve of the native corneal endothelium. The current status, biological maturity, and translational readiness of iPSC-derived corneal cell types are summarized in Table 1.

### 3.3. Mechanistic Basis of iPSC-Mediated Regeneration

Beyond direct cellular replacement, iPSC-derived corneal cells may exert important paracrine and immunomodulatory effects. Proposed mechanisms include modulation of local inflammatory responses, attenuation of profibrotic signaling pathways, promotion of extracellular matrix remodeling, and enhancement of tissue repair processes. Increasing evidence suggests that extracellular vesicles, exosomes, and secreted bioactive factors may contribute substantially to these regenerative effects, raising interest in the development of cell-free therapeutic approaches. However, the relative contribution of direct engraftment versus paracrine signaling remains incompletely understood and likely varies across corneal compartments and disease models.

## 4. Preclinical Evidence

### 4.1. iPSC-Derived Corneal Epithelium

Preclinical research on iPSC-derived corneal epithelium has progressed substantially over the past decade, evolving from exploratory differentiation experiments toward translational animal models aimed at restoring ocular surface homeostasis in limbal stem cell deficiency (LSCD). Early studies primarily focused on determining whether pluripotent cells could be directed toward a corneal epithelial lineage. More recent work has shifted toward improving biological fidelity, epithelial maturation, regenerative hierarchy, graft integration, and long-term safety following transplantation [7,12,13]. In parallel, several groups have refined differentiation protocols and explored transdifferentiation strategies to improve lineage specificity, reproducibility, and therapeutic consistency across iPSC lines [9,14,15].

Protocols established by Hayashi and colleagues represented a major advance in the field by recapitulating developmental signaling pathways involved in ocular surface specification [7,12]. These approaches generated stratified epithelial sheets expressing corneal epithelial markers such as CK3/12 while retaining a basal cell population positive for stem/progenitor-associated markers including p63α and ABCG2. The preservation of this progenitor-like compartment is biologically important, as long-term epithelial maintenance depends not only on differentiated surface cells but also on sustained regenerative capacity. In rabbit LSCD models, transplantation of engineered epithelial sheets resulted in restoration of epithelial coverage, organized multilayer formation, and partial suppression of conjunctivalization [7,12]. Histological analyses further demonstrated stable attachment to the host stroma and relatively preserved epithelial architecture, supporting the concept that iPSC-derived epithelial constructs may provide more than temporary surface replacement. However, differences in differentiation efficiency and marker expression across protocols continue to complicate reproducibility and direct inter-study comparison. Recent studies suggest that optimized culture conditions and small-molecule signaling modulation may partially reduce this variability and improve epithelial specification consistency [14,15].

A major advance in translational validation was demonstrated by Yoshinaga et al. (2022) [13], who extended preclinical assessment in non-human primate models. Human iPSC-derived corneal epithelial sheets transplanted onto experimentally induced LSCD corneas showed stable integration, restored multilayered epithelial architecture, and maintained suppression of conjunctival overgrowth. Importantly, longitudinal follow-up revealed no dysplastic changes, uncontrolled proliferation, or teratoma formation, addressing central safety concerns associated with pluripotent stem cell–based therapies. Minimal immune-mediated rejection further suggests that the ocular surface microenvironment, together with appropriate differentiation strategies, can help mitigate immunogenicity in this setting. Recent preclinical evaluations have also incorporated organoid-based or 3D culture systems to better mimic native limbal niche conditions, offering additional mechanistic insight into cell–cell interactions and tissue organization [14,15].

Beyond two-dimensional epithelial sheet transplantation, three-dimensional differentiation and organoid-based systems derived from iPSCs have provided additional mechanistic insights [16,17]. These models exhibit self-organizing epithelial domains and transcriptional signatures closely resembling native limbal epithelial stem cells. Gene expression analyses demonstrate convergence toward limbal-associated molecular programs, reinforcing that directed differentiation recapitulates developmental lineage specification rather than mere superficial phenotypic mimicry.

Across preclinical models—including rodents, rabbits, and non-human primates—tumorigenicity has not been observed when fully differentiated epithelial sheets devoid of residual pluripotent markers are used [7,12,13]. Cytogenetic stability and absence of OCT4 or NANOG expression at the time of grafting have been consistently emphasized, highlighting the importance of rigorous quality control prior to clinical translation. Nonetheless, extended observation under chronic inflammatory conditions remains necessary to fully establish long-term safety margins. Recent preclinical studies also underscore the importance of monitoring under inflammatory stress and immunologically challenging conditions to confirm robust engraftment [9,14].

Collectively, current preclinical evidence supports the ability of iPSC-derived corneal epithelium to form stratified, functionally organized tissue, restore ocular surface integrity in LSCD models, and maintain short- to mid-term safety. While differentiation efficiency and inter-line variability remain challenges, these data position iPSC-derived epithelium as a scalable, donor-independent alternative to conventional limbal transplantation techniques.

### 4.2. iPSC-Derived Corneal Stroma

Recent in vitro studies have established increasingly effective protocols for directing human induced pluripotent stem cells (iPSCs) toward a keratocyte-like phenotype. In a landmark study, Chen and colleagues developed an embryoid body (EB)-based differentiation approach using chemically defined culture conditions, generating keratocyte-like cells that expressed characteristic stromal markers including aldehyde dehydrogenase 1 family member A1 (ALDH1A1), lumican, and keratocan, while demonstrating minimal expression of fibroblastic or myofibroblastic markers [11]. This distinction is biologically important because maintenance of a stable keratocyte phenotype is essential for preserving stromal transparency and minimizing fibrotic remodeling, both of which remain major challenges in corneal stromal regeneration.

Despite substantial progress in in vitro differentiation, direct in vivo evidence supporting functional stromal regeneration using iPSC-derived keratocytes remains limited. To date, few studies have demonstrated successful long-term transplantation of iPSC-derived keratocyte-like cells with stable integration into host stroma and restoration of native extracellular matrix (ECM) architecture. This limitation reflects the exceptional structural complexity of the corneal stroma, in which precise collagen lamellar organization and stromal ultrastructure are critical for optical transparency yet remain difficult to reproduce ex vivo or through current tissue-engineering strategies [18]. Consequently, the translation of molecular keratocyte differentiation into functional stromal tissue reconstruction remains incompletely resolved.

Related preclinical studies using alternative stem cell populations have nevertheless provided insight into potential regenerative mechanisms relevant to stromal repair. Mesenchymal stem cells (MSCs), derived from multiple tissue sources, have demonstrated partial differentiation toward keratocyte-like phenotypes in animal models and have been associated with stromal remodeling, extracellular matrix deposition, and reduced inflammatory responses following corneal injury. In some studies, MSC-based therapies promoted secretion of stromal ECM components, including type I collagen, while reducing scar formation and stromal haze [19]. These findings suggest that stromal regeneration may depend not only on direct cellular replacement but also on paracrine modulation of the wound-healing microenvironment, which may have implications for future iPSC-based approaches.

In parallel, emerging studies investigating exosome-based therapies derived from iPSC-associated mesenchymal populations have reported preliminary antifibrotic and wound-modulating effects in experimental corneal injury models. Proposed mechanisms include promotion of native stromal cell proliferation, modulation of inflammatory signaling pathways, and reduction in profibrotic gene expression [20]. However, these approaches remain indirect regenerative strategies and do not yet demonstrate reconstruction of native stromal architecture through durable keratocyte engraftment.

Collectively, current evidence supports the feasibility of generating keratocyte-like cells from iPSCs under controlled in vitro conditions. However, stromal regeneration remains substantially less advanced than epithelial or endothelial applications, particularly with respect to long-term in vivo integration, restoration of native collagen organization, and maintenance of optical transparency. Further progress will likely require improved differentiation stability, advanced scaffold and bioengineering strategies, and rigorous long-term evaluation in clinically relevant animal models before meaningful translational application can be achieved.

### 4.3. iPSC-Derived Corneal Endothelium

**Figure 2:**
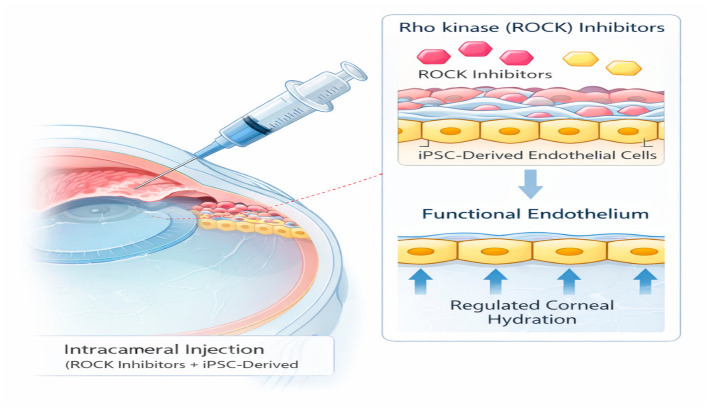
Schematic representation of intracameral delivery of iPSC-derived corneal endothelial cells combined with Rho-associated kinase (ROCK) inhibition for endothelial regeneration. ROCK inhibition enhances endothelial cell adhesion, survival, and functional integration following transplantation.

Foundational preclinical studies demonstrated that iPSC-derived corneal endothelial cells (CECs) can acquire characteristic hexagonal morphology and express key functional markers, including Na^+^/K^+^-ATPase and tight junction-associated proteins. In non-human primate models of endothelial dysfunction, transplanted iPSC-derived CECs integrated into the host posterior cornea, restored corneal transparency, reduced stromal edema, and partially recovered endothelial pump and barrier functions [21]. Immunohistochemical analyses further confirmed the presence of human-specific endothelial markers within regenerated tissue, supporting successful cellular engraftment. Notably, intracameral delivery of iPSC-derived endothelial cells combined with Rho-associated kinase (ROCK) inhibition has been associated with sustained corneal clearing and functional recovery for follow-up periods extending up to 24 weeks in preclinical models. A schematic representation of intracameral delivery of iPSC-derived corneal endothelial cells combined with ROCK inhibition is shown in Figure 2.

Similar findings have been reported in rabbit models of endothelial injury, where iPSC-derived CECs differentiated through corneal endothelial progenitor-like intermediates (CEXI) were administered via intracameral injection [7,22]. These studies demonstrated improvement in corneal clarity, reduction in stromal swelling, and integration of endothelial marker-positive cells along the posterior corneal surface. Importantly, no uncontrolled proliferation or tumor formation was observed during the reported follow-up periods, providing preliminary support for the safety of differentiated endothelial cell transplantation. However, most available studies remain limited by relatively short observation periods and variability in differentiation protocols, making long-term functional durability difficult to assess.

To improve endothelial cell survival and engraftment efficiency, several tissue-engineering strategies incorporating biomaterial scaffolds and thermosensitive hydrogels have also been explored. In experimental rabbit models, hydrogel-assisted transplantation improved endothelial cell density and corneal transparency compared with cell injection alone, suggesting that microenvironmental support may play an important role in maintaining endothelial homeostasis after transplantation [23]. These findings further highlight the importance of extracellular matrix interactions and biomechanical support in endothelial regeneration.

Investigations involving iPSC-derived corneal endothelial progenitor-like cells (CEXI) have additionally suggested the potential for temporary restoration of corneal hydration and thickness regulation. Nevertheless, incomplete endothelial maturation, phenotypic instability, and endothelial-to-mesenchymal transition remain important concerns that may compromise long-term functional performance following transplantation [24]. These observations emphasize the need for standardized differentiation protocols, rigorous quality-control measures, and reliable functional assays before broader clinical translation can be achieved.

Beyond transplantation-based approaches, three-dimensional corneal organoid systems derived from iPSCs have emerged as valuable platforms for mechanistic and translational research. These organoids can partially recapitulate epithelial, stromal, and endothelial compartment organization, enabling investigation of cell–cell interactions, developmental signaling pathways, drug responses, and disease modeling in controlled experimental settings that are difficult to reproduce in vivo [16]. However, organoid systems still demonstrate variability in structural organization and functional maturity, limiting their immediate therapeutic applicability.

Collectively, current preclinical evidence indicates that iPSC-derived corneal endothelial cells can partially restore endothelial function and corneal clarity in experimental models of endothelial dysfunction. Nevertheless, important challenges remain regarding long-term graft stability, endothelial maturation, immunologic safety, and maintenance of durable pump function following transplantation. Continued optimization of differentiation methods, delivery systems, and long-term in vivo evaluation will therefore be essential before widespread clinical application becomes feasible.

## 5. Clinical Translation and Human Studies

Clinical translation of iPSC-based corneal therapies remains at an early stage, although the most advanced progress to date has been observed in the treatment of limbal stem cell deficiency (LSCD). In Japan, pioneering first-in-human studies evaluated transplantation of iPSC-derived corneal epithelial sheets (iCEPS) in patients with severe bilateral LSCD (*n* = 4) with a follow-up extending up to 24 months, representing one of the earliest clinical applications of iPSC-based ocular surface regeneration [10]. These epithelial sheets were generated under clinical-grade manufacturing conditions and transplanted onto the ocular surface following removal of fibrovascular and pathologic tissue.

During follow-up periods extending up to two years, treated eyes demonstrated sustained epithelial coverage, reduction in corneal neovascularization, and improvement in visual acuity and ocular surface stability. Reported adverse events were generally mild and manageable, and no tumor formation or severe proliferative complications were identified during the observation period. These findings provided important preliminary evidence supporting the short- to mid-term feasibility and safety of iPSC-derived epithelial transplantation. At the same time, the study highlighted several practical translational challenges, including maintenance of genomic stability during cell expansion, quality-control standardization, large-scale manufacturing consistency, and optimization of surgical handling to improve engraftment reliability.

Despite these encouraging findings, current evidence remains limited by small sample size, absence of control groups, and relatively short follow-up duration. Consequently, larger prospective studies will be required to determine long-term graft durability, reproducibility of functional outcomes, cost-effectiveness, and comparative efficacy relative to established therapies [10]. Current treatment strategies for LSCD primarily focus on restoration of the limbal stem cell niche and stabilization of the ocular surface. These include autologous procedures such as conjunctival limbal autograft (CLAU) and simple limbal epithelial transplantation (SLET), which avoid systemic immunosuppression but are limited to unilateral disease. Additional approaches include cultivated limbal epithelial transplantation (CLET), allogeneic keratolimbal transplantation, and keratoprosthesis in advanced or refractory cases. Although these techniques have demonstrated meaningful clinical benefit, they remain constrained by donor tissue availability, surgical complexity, immune rejection, and long-term maintenance requirements. iPSC-derived epithelial therapies therefore represent a potentially scalable and donor-independent alternative, although low-dose perioperative immunosuppression was still required in two of the four patients in the first-in-human study. A comparative overview of current treatment strategies for limbal stem cell deficiency is presented in Table 2.

Beyond epithelial applications, iPSC-derived corneal endothelial therapies have also entered first-in-human clinical investigation, representing another important milestone in translational corneal regeneration [25]. Early clinical studies have focused primarily on evaluating safety, cellular survival, and restoration of endothelial function following intracameral delivery of endothelial-like cells. In the initial first-in-human study, an allogeneic iPSC-derived endothelial substitute (CLS001) was administered into the anterior chamber of a single patient (*n* = 1) with one-year postoperative follow-up, allowing transplanted cells to attach to the posterior corneal surface. Clinical monitoring included assessment of corneal transparency, stromal thickness, endothelial cell density, and adverse events over time.

During one year of follow-up, the treated eye demonstrated improvement in visual acuity and reduction in corneal edema without clear evidence of immune rejection or major safety-related complications. However, genomic analysis identified a de novo in-frame deletion involving the *EP300* gene within the transplanted cell population. Importantly, this alteration was absent in both the original donor cells and the master clinical-grade cell bank, suggesting acquisition during in vitro expansion. Although no clinically evident adverse effects were associated with this finding during follow-up, it highlights a central challenge in iPSC-based therapy development: the potential emergence of genomic alterations during prolonged culture and manufacturing processes. These observations underscore the importance of comprehensive genomic surveillance, standardized release criteria, and long-term post-transplant monitoring for all iPSC-derived clinical products.

Although clinical experience remains extremely limited, these early studies provide important insight into the feasibility, safety considerations, and translational complexity of iPSC-derived corneal therapies in humans. At present, however, evidence remains insufficient to determine long-term functional durability, comparative clinical benefit, immunologic behavior, or economic feasibility relative to existing treatment approaches. Larger multicenter studies with prolonged follow-up will therefore be essential before routine clinical adoption can be considered.

## 6. Challenges and Limitations

Several major challenges continue to limit the widespread clinical translation of iPSC-based corneal therapies. One of the principal concerns remains the potential tumorigenic risk associated with residual undifferentiated pluripotent cells, necessitating rigorous purification strategies and comprehensive quality-control measures prior to transplantation. In practice, quality assurance extends beyond assessment of lineage-specific marker expression and includes evaluation of cellular identity, differentiation efficiency, genomic and epigenetic stability, sterility, functional maturity, and exclusion of residual pluripotent populations. The establishment of standardized release criteria and validated potency assays will therefore be essential to ensure manufacturing consistency, reproducibility, and clinical safety across production batches.

Immunogenicity also remains incompletely understood. Although autologous iPSC-derived therapies were initially expected to minimize immune rejection, both preclinical and early clinical observations suggest that immune responses may still occur, potentially related to genetic and epigenetic alterations acquired during cellular reprogramming, prolonged culture, or differentiation processes [26,27]. In addition, substantial inter-line variability continues to complicate reproducibility, scalability, and comparative safety assessment. Differences in donor source, reprogramming technique, and culture conditions may all influence differentiation behavior and long-term cellular stability.

Genomic instability represents another major translational concern in iPSC-derived corneal therapies. Genetic alterations may pre-exist within donor somatic cells, emerge during cellular reprogramming, or accumulate progressively during prolonged in vitro expansion and passaging. Importantly, these abnormalities may not always be detectable through routine phenotypic characterization alone and may influence long-term cellular behavior, differentiation fidelity, phenotypic stability, immunogenicity, or tumorigenic potential.

The recent identification of an *EP300* mutation in a first-in-human endothelial study further underscores the clinical relevance of continuous genomic surveillance during manufacturing and post-transplant monitoring. Although no mutation-related adverse clinical events were observed during follow-up, this finding highlights that even extensively characterized iPSC-derived products may retain residual biological uncertainty requiring ongoing evaluation.

Beyond isolated gene mutations such as *EP300*, the broader implications of genomic instability extend to concerns regarding reproducibility, long-term biological behavior, and regulatory reliability. Accumulation of subclonal genetic alterations during prolonged culture may generate heterogeneous cellular populations with altered differentiation trajectories, phenotypic instability, or selective growth advantages that may not become clinically evident until long after transplantation. Such variability may also compromise batch-to-batch consistency and complicate GMP-compliant manufacturing and standardization across independently generated iPSC lines.

Furthermore, because some genomic abnormalities may remain biologically silent during early follow-up, the absence of immediate adverse events does not eliminate the possibility of delayed phenotypic instability, loss of functional maturity, immune dysregulation, or tumorigenic transformation. These concerns highlight the importance of comprehensive genomic surveillance strategies—including whole-genome sequencing, copy number variation analysis, validated release criteria, and long-term post-transplant monitoring—within future clinical translation frameworks for iPSC-derived corneal therapies.

Beyond biological concerns, large-scale good manufacturing practice (GMP)-compliant production remains technically complex and economically demanding. Standardization of culture conditions, automation of manufacturing workflows, cryopreservation stability, transportation logistics, and regulatory harmonization all remain unresolved challenges for broader clinical implementation [9]. In addition, the individualized nature of autologous iPSC generation may limit scalability and increase production costs compared with conventional donor-based therapies.

To address some of these limitations, centralized iPSC-derived corneal cell banking systems have been proposed as a potential strategy for improving standardization and accessibility [9,25,28]. Such platforms may facilitate standardized genomic screening, HLA matching, traceability, cryopreservation, and quality-control oversight under GMP conditions. However, important logistical, regulatory, ethical, and economic challenges remain regarding long-term storage, large-scale implementation, international distribution, and maintenance of consistent quality standards across institutions.

**Figure 3:**
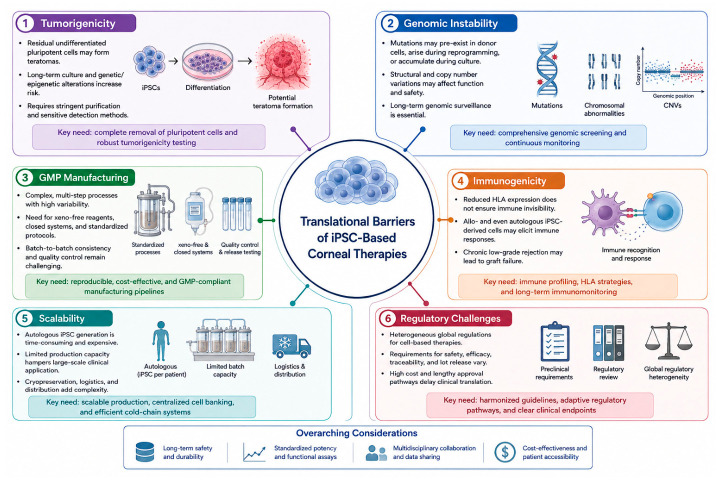
Major translational barriers limiting clinical implementation of iPSC-based corneal regenerative therapies, including tumorigenicity, genomic instability, immunogenicity, GMP-compliant manufacturing complexity, scalability, and regulatory considerations.

Collectively, these challenges highlight that although iPSC-based corneal regeneration remains highly promising, substantial biological, manufacturing, regulatory, and economic barriers must still be addressed before routine clinical adoption can become feasible. The major translational barriers and challenges associated with clinical implementation of iPSC-derived corneal therapies are summarized in Figure 3.

## 7. Expert Perspective and Critical Appraisal from a Clinical–Translational Standpoint

From a clinical–translational perspective, current evidence suggests that the major barriers to iPSC-based corneal regeneration are shifting from proof-of-concept feasibility toward issues related to safety, standardization, scalability, and long-term clinical implementation. Considerable progress has been achieved across epithelial, stromal, and endothelial applications. However, each corneal lineage presents distinct biological and translational challenges related to differentiation efficiency, functional maturation, tissue integration, and long-term phenotypic stability. These factors substantially influence both safety considerations and the pace of clinical translation.

Among currently investigated applications, iPSC-derived corneal epithelium appears to demonstrate the most advanced translational readiness. Epithelial constructs exhibit regenerative capacity, physiological cellular turnover, and relative accessibility for direct clinical monitoring following transplantation. In addition, epithelial grafts may theoretically offer a more favorable safety profile because adverse outcomes can potentially be detected early and managed through surgical removal or replacement. These characteristics may partially explain why epithelial applications, particularly for bilateral limbal stem cell deficiency, have progressed most rapidly toward first-in-human clinical studies. Nevertheless, current clinical evidence remains limited, and long-term durability, reproducibility, and comparative efficacy relative to established limbal transplantation techniques have yet to be fully established.

By comparison, iPSC-derived corneal endothelial therapies may represent one of the most clinically impactful yet biologically demanding regenerative applications in ophthalmology. Because endothelial dysfunction remains a leading indication for corneal transplantation worldwide, successful restoration of endothelial cell density and pump function through cell-based therapy could substantially reduce dependence on donor tissue. Encouraging preclinical findings, including studies using non-human primate models and intracameral delivery strategies combined with Rho-associated kinase (ROCK) inhibition, have demonstrated partial recovery of corneal clarity and endothelial function. However, endothelial applications are also associated with narrower safety margins because even limited cell loss, phenotypic instability, or functional decline may have clinically significant consequences. Concerns related to residual pluripotent cells, genomic instability, endothelial-to-mesenchymal transition, and long-term graft durability therefore remain particularly important in this setting.

Immunogenicity further complicates the translational landscape. Although iPSC-derived products are often considered immunologically advantageous, reduced HLA expression does not eliminate the possibility of immune-mediated responses. Even low-grade chronic endothelial cell loss may ultimately compromise graft survival and long-term function. Consequently, several groups have proposed partially HLA-matched or universal donor iPSC banking systems as potentially more practical and scalable alternatives to fully individualized autologous approaches, which remain resource-intensive and logistically challenging. However, the long-term immunologic behavior of these strategies remains incompletely characterized.

In addition to biological limitations, manufacturing and regulatory considerations remain major rate-limiting factors for clinical implementation. Reproducible GMP-compliant differentiation protocols, standardized release criteria, exclusion of residual undifferentiated cells, long-term genomic surveillance, and structured post-transplant monitoring frameworks will all be essential for broader clinical adoption. Differences in regulatory infrastructure across countries may also partly explain why early iPSC clinical trials have been concentrated within a limited number of specialized centers with centralized manufacturing capabilities and adaptive translational pathways. Key translational characteristics, advantages, and limitations of iPSC-derived corneal therapies are summarized in Table 3.

Collectively, current evidence indicates that iPSC-based corneal regeneration has progressed from theoretical feasibility toward early translational application. However, human clinical experience remains extremely limited, and long-term safety, immunologic stability, manufacturing scalability, regulatory harmonization, and economic feasibility remain unresolved. Future progress in the field will therefore depend not only on biological innovation, but also on the development of reproducible manufacturing systems, rigorous long-term clinical evaluation, and internationally consistent regulatory frameworks.

## 8. Future Perspectives

Future progress in iPSC-based corneal regeneration will likely depend on continued advances in gene-editing technologies, bioengineering platforms, and large-scale manufacturing systems. In particular, CRISPR/Cas9-mediated approaches aimed at generating hypoimmunogenic or partially HLA-matched universal iPSC lines may help reduce immunologic barriers and improve scalability for broader clinical use [29]. However, the long-term genomic safety and regulatory implications of gene-edited cellular products remain incompletely understood and will require careful evaluation.

Additional progress is expected through integration of iPSC-derived cells with biomaterial scaffolds, optimization of tissue-engineered corneal constructs, and refinement of three-dimensional organoid systems capable of more closely reproducing native corneal architecture and microenvironmental signaling [9]. Advances in automated manufacturing workflows, cryopreservation protocols, and standardized quality-control frameworks may further improve reproducibility and facilitate multicenter clinical implementation.

Centralized iPSC banking systems and coordinated national translational programs, particularly those established in Japan, may also provide important regulatory and infrastructural models for future international development. Nevertheless, successful global implementation will likely require harmonization of regulatory standards, long-term post-transplant surveillance strategies, and demonstration of cost-effectiveness relative to existing therapies.

As the field continues to evolve, future research will need to focus not only on improving biological efficacy, but also on addressing durability, scalability, accessibility, and long-term safety in clinically relevant settings.

## 9. Conclusions

Induced pluripotent stem cells represent a versatile platform for corneal regenerative medicine, with the capacity to generate epithelial, stromal, and endothelial cell populations from a renewable cellular source. Over the past decade, substantial progress has been achieved in differentiation strategies, preclinical modeling, and early translational studies, particularly in the treatment of limbal stem cell deficiency and endothelial dysfunction.

Despite these advances, important biological and translational barriers remain unresolved, including genomic instability, immunogenicity, phenotypic variability, manufacturing complexity, regulatory standardization, and long-term functional durability following transplantation. In addition, the degree of evidence currently available differs substantially across corneal compartments, with stromal regeneration remaining comparatively underdeveloped relative to epithelial and endothelial applications.

Current preclinical and early clinical findings nevertheless support the therapeutic potential of iPSC-based corneal regeneration and justify continued investigation in carefully designed translational studies. Future progress will depend on rigorous long-term safety assessment, standardized manufacturing pipelines, multicenter clinical validation, and development of scalable regulatory frameworks capable of supporting broader clinical implementation.

## Figures and Tables

**Table 1:** Status of iPSC-Derived Corneal Cell Types: Biological Maturity and Translational Readiness [7,8,9,11].

Corneal Layer	iPSC-Derived Cell Type	Key Functional Properties	Representative Quantitative/Translational Findings	Preclinical Evidence	Clinical Status	Principal Translational Challenges
Epithelium	Corneal epithelial-like cells	Stratification, barrier function, KRT3/KRT12 expression	Stable epithelialization and ocular surface improvement reported with follow-up up to 24 months in first-in-human studies	Strong	Early first-in-human studies	Manufacturing cost, scalability, long-term epithelial stability
Stroma	Keratocyte-like cells	Extracellular matrix deposition, reduced profibrotic phenotype	Expression of stromal markers including ALDH1A1, keratocan, and lumican; limited convincing long-term in vivo integration data	Moderate	Preclinical only	Phenotypic instability, limited in vivo stromal integration, ECM organization challenges
Endothelium	Endothelial-like cells	Pump-associated gene expression, barrier integrity	Reduction in corneal edema and partial restoration of transparency reported in preclinical and early human studies; follow-up up to 12 months	Strong	Early first-in-human studies	Safety, immunogenicity, long-term functional durability

Table 1 Legend. Among iPSC-derived corneal cell populations, epithelial constructs currently appear closest to broader clinical application. In contrast, stromal regeneration remains insufficiently validated in vivo, whereas endothelial regeneration represents a highly promising but technically and biologically demanding translational target.

**Table 2:** Comparative Overview of Current LSCD Therapies.

Therapy	Cell Source	Key Limitations	Immunosuppression
SLET (Simple Limbal Epithelial Transplantation)	Autologous limbal tissue from fellow eye	Dependent on residual healthy limbal tissue; variable outcomes in severe ocular surface inflammation	Not required
CLAU (Conjunctival Limbal Autograft)	Autologous limbal graft from fellow eye	Limited to unilateral disease; potential donor-site morbidity; limited tissue availability	Not required
CLET (Cultivated Limbal Epithelial Transplantation)	Ex vivo expanded limbal epithelial cells	High manufacturing cost; GMP complexity; variability in cell quality; specialized laboratory requirements	Required in allogeneic settings
KLAL/lr-CLAL	Allogeneic donor limbal tissue	Risk of immune rejection and graft failure; long-term systemic immunosuppression	Required
Keratoprosthesis	Artificial corneal implant	Device-related complications; glaucoma; extrusion risk; melting risk; intensive follow-up	Variable

Table 2 Legend. Current LSCD therapies can provide meaningful ocular surface restoration but remain limited by donor tissue dependence, immune-related complications, surgical complexity, and long-term maintenance.

**Table 3:** Comparative Translational Characteristics of iPSC-Derived Corneal Epithelial and Endothelial Therapies.

Translational Parameter	Corneal Epithelium	Corneal Endothelium
Cellular turnover	High	Minimal
Functional reserve	Relatively preserved	Limited
Clinical monitoring	Direct visualization	Primarily indirect
Consequences of partial cell loss	Clinically apparent	Clinically apparent
Near-term clinical readiness	More advanced	Early translational stage
Major translational concern	Long-term epithelial stability	Functional durability and safety

Table 3 Legend. Although both epithelial and endothelial iPSC-derived therapies demonstrate substantial regenerative potential, endothelial applications currently present narrower safety margins and greater translational complexity, necessitating more rigorous long-term evaluation before routine clinical implementation.

## Data Availability

No new data were created or analyzed in this review study. Data sharing is not applicable.
